# Aged iPSCs display an uncommon mitochondrial appearance and fail to undergo in vitro neurogenesis

**DOI:** 10.18632/aging.100708

**Published:** 2014-12-30

**Authors:** Andrea Masotti, Antonella Celluzzi, Stefania Petrini, Enrico Bertini, Ginevra Zanni, Claudia Compagnucci

**Affiliations:** ^1^ Gene Expression–Microarrays Laboratory, Bambino Gesù Children's Hospital, IRCCS, 00165 Rome, Italy; ^2^ Confocal Microscopy Core Facility, Research Laboratories, Bambino Gesù Children's Hospital, IRCCS, Rome 00165, Italy; ^3^ Unit of Neuromuscular and Neurodegenerative Disorders, Laboratory of Molecular Medicine, Department of Neurosciences, Bambino Gesù Children's Research Hospital, IRCCS, Piazza S. Onofrio, 4 00165 Rome, Italy

**Keywords:** induced pluripotent stem cells, mitochondria, stem cell aging, mitochondrial dysfunction, in vitro neurogenesis

## Abstract

Reprogramming of human fibroblasts into induced pluripotent stem cells (iPSCs) leads to mitochondrial rejuvenation, making iPSCs a candidate model to study the mitochondrial biology during stemness and differentiation. At present, it is generally accepted that iPSCs can be maintained and propagated indefinitely in culture, but no specific studies have addressed this issue. In our study, we investigated features related to the 'biological age' of iPSCs, culturing and analyzing iPSCs kept for prolonged periods *in vitro*. We have demonstrated that aged iPSCs present an increased number of mitochondria per cell with an altered mitochondrial membrane potential and fail to properly undergo *in vitro* neurogenesis. In aged iPSCs we have also found an altered expression of genes relevant to mitochondria biogenesis. Overall, our results shed light on the mitochondrial biology of young and aged iPSCs and explore how an altered mitochondrial status may influence neuronal differentiation. Our work suggests to deepen the understanding of the iPSCs biology before considering their use in clinical applications.

## INTRODUCTION

Induced pluripotent stem cells (iPSCs) are stem cells reprogrammed from adult somatic cells of different embryonic origin such as the ectoderm (i.e., keratinocytes and neural cells), the endoderm (i.e., hepatocytes) and the mesoderm (i.e., fibroblasts) [1]. The great potential of iPSCs consists in providing cell sources for better understanding various diseases, for screening drugs, and for performing cell transplantation therapies. But, before iPSC technology can be considered as an effective tool for translational medicine, many challenges should be overcome. Following reprogramming, propagation of iPSCs is necessary to obtain the number of iPSCs sufficient to perform characterizations of the derived clone, downstream investigations and applications. At present the concept that iPSCs can be maintained and propagated indefinitely in culture is broadly accepted [2, 3, 4], despite during iPSC long-term culturing, the epigenetic status of the cells may change and their tumorigenic potential is increased [5]. Even if iPSC-technology has allowed '*in vitro* disease modeling' of several (still poorly known) diseases and, importantly iPSCs have the potential to be used for self-transplantation, great control and responsibility must be taken in their usage. In fact, the mechanisms of iPSC aging and its opposite (rejuvenation) during somatic cell reprogramming are mostly unknown and finding features that efficiently measure age *in vitro* is one purpose of this project. A deeper understanding of the molecular determinants placed in the local niche and controlling self-renewal versus differentiation is needed. Importantly, the ability to recreate the correct stem cell niche *in vitro* is lacking and this hinders studying iPSCs or expanding them for therapy. At present stem cell aging *in vivo* is considered a consequence of an altered stem cell niche, where local intercellular signals changes and the stem cell environment becomes aged [6]. Currently, great attention has been given to the understanding of iPSC reprogramming and, in fact, it is well established that iPSCs can rely on a rejuvenated state capable of escaping cellular senescence. In this work, we have investigated the iPSCs biology of aging, focusing in particular on the mitochondrial endowment in relation to short- *versus* long-term maintenance of iPSCs in culture. Many studies have demonstrated that iPSCs are very similar to embryonic stem cells (ESCs) in terms of pluripotency and differentiation potential [7, 8]. iPSCs generated from senescent cells have reset gene expression profiles and mitochondrial metabolism, resulting indistinguishable from ESCs and maintaining the ability to re-differentiate into fully rejuvenated cells [9]. Importantly, the iPSCs employed in this study have been obtained using the episomal ‘integration-free’ non-viral technology. This technique has a lower efficiency when compared to the lentiviral reprogramming method used by Lapasset et al. [9]. Notwithstanding, it allows to study phenotypes without the problematic issue of genomic random integration, which may perturb the sequence of relevant genes as those implicated in processes regulating pluripotency/differentiation/metabolism. Other authors have investigated whether iPSCs present signs of cellular rejuvenation similarly to ESCs [10, 11, 12]. In line with these studies, focused on telomere elongation, the characterization of the structural and functional properties of mitochondria in iPSCs demonstrated that cell reprogramming also rejuvenates mitochondria similarly to what observed in ESCs [13, 14]. In fact, the morphology, localization, abundance and function of mitochondria are suggested to represent markers of pluripotency [15]. The main characteristics of iPSCs and ESCs mitochondria are their round-shaped morphology with condensed cristae and their poor oxidative activity due to the low membrane potential (e.g. when compared with that of teratoma-derived fibroblasts) [13, 16]. ESCs and iPSCs contain few mitochondria that progressively increase in number during differentiation, when the cell undergoes different and more energy-demanding activities [17, 18]. In fact, cellular differentiation requires a metabolic switch from glycolysis to oxidative phosphorylation and mitochondria are necessary to this biological function [19]. This switch also involves the activation of some crucial factors/genes that determine specific changes during development and aging [20]. A recent study on iPSCs with a heavy mitochondrial DNA mutation load demonstrates the differential requirements of mitochondrial integrity for pluripotent stem cell self-renewal versus differentiation, and highlights the relevance of assessing the integrity of the mitochondrial genome when aiming to generate iPSCs cells with robust differentiation potential [21]. Moreover, mice with mutator mtDNA (due to a error-prone replication of mtDNA due to a dysfunctional Polg) acquire premature aging phenotypes including weight loss, osteoporosis, anemia and reduced life spans [22]. Overall, these data suggest that mitochondria have a crucial role in the physiological balance between pluripotency and differentiation and, importantly, they allow us to discuss on the aging biology of iPSCs, which is a poorly understood topic in need of research attention.

## RESULTS

**Figure 1:**
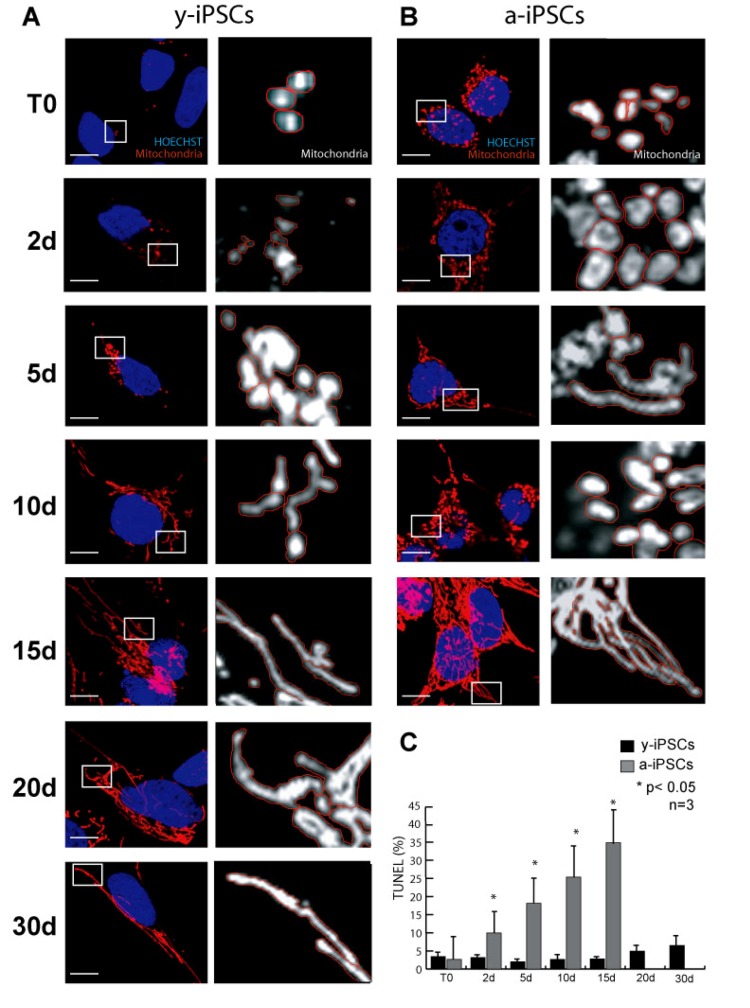
Characterization of mitochondria in y- and a-iPSCs in proliferating condition and during *in vitro* neurogenesis (**A, B**) Immunofluorescence analysis of mitochondria (red) in y-iPSCs (**A**) and in a-iPSCs (**B**) with nuclei counterstained with Hoechst (blue). On the right side of each photograph a higher magnification of the mitochondria is reported, mitochondria are colored in white and the perimeter is highlighted in red for better morphological visualization. Scale bar: 10 μm. (**C**) Quantification of the TUNEL assay in y- and a-iPSCs before and during neuronal differentiation. The bar graph represents quantitative data (expressed in % of TUNEL assay positive cells) of y-iPSCs and a-iPSCs. Data represent the mean ± SD of 3 experiments.

**Figure 2:**
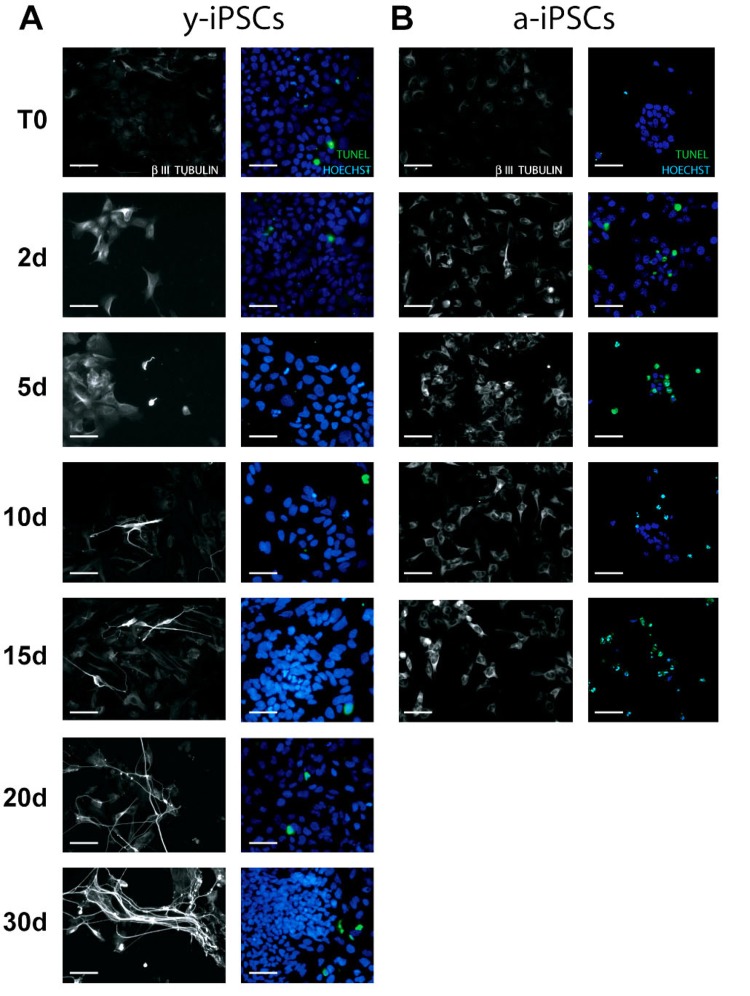
Analysis of neuronal differentiation and cell death in y- and a-iPSCs Immunofluorescence analysis of y-iPSCs (**A**) and a-iPSCs (**B**) during neuronal differentiation stained with the neuronal marker β–III TUBULIN with nuclei counterstained with Hoechst. On the right of each photographs, the TUNEL assay corresponding to the same experiment is reported. The green cells represent TUNEL positive cells. Scale bar: 30 μm.

**Figure 3:**
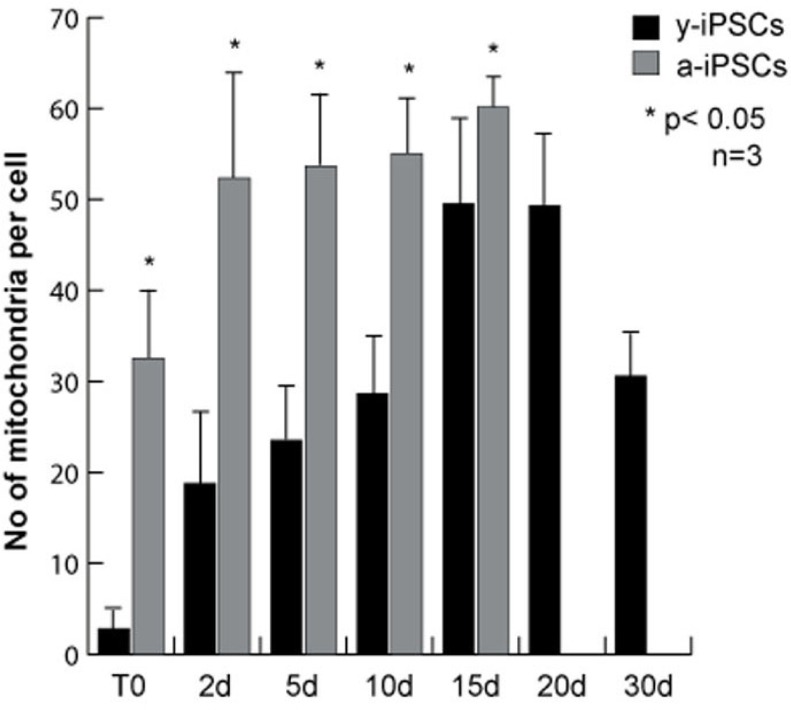
Quantitative analysis of the number of mitochondria in y- and a-iPSCs before and during neuronal differentiation The bar graph represents the average number of mitochondria per cell and was obtained counting the mitochondria from immunofluorescence assays using the mitochondria specific antibody. The data were obtained from three independent experiments. Data are represented as mean ± SD.

To study the morphology and the number of iPSC mitochondria, we performed immunofluorescence assays for α-mitochondria on young iPSCs (named y-iPSCs) kept in culture for 1 month after reprogramming, and on aged iPSCs (named a-iPSCs) that have been cultured for more than one year (Fig. 1 and 2). We observed that y-iPSCs have a reduced number of mitochondria (2.85 ± 1.43 per cell) compared to a-iPSCs (32.55 ± 9.90 per cell) (Fig. 1A, and 3). These data suggest that long-term maintenance of iPSCs in culture may alter the number of mitochondria.

Having established that a-iPSCs present alterations of their mitochondrial number, we investigated whether this cellular alteration compromises the ability of iPSCs to differentiate. Since the balance between cell proliferation and differentiation relies on the mitochondrial state of the cell, we compared the mitochondrial development and the differentiation potential of y- and a-iPSCs. With this aim and considering that the neuron is the cell with the highest number of mitochondria [23], we differentiated iPSCs into motor neurons (Fig. 2). In optimal conditions (chemically defined differentiating medium), 95-99% of iPSCs differentiate into motor neurons after 30 days. The main features of mature neurons are the positivity to the neuronal marker β-III-TUBULIN and the formation of elongated and branched neurites (Fig. 2). At the beginning of *in vitro* neurogenesis (T0), mitochondria of y-iPSCs were few and had a rounded shape but they increased in number and acquired the elongated and mature morphology of an organized mitochondrial network from day 10 (10d) to day 30 (30d) of differentiation (Fig. 1A). At T0, mitochondria of a-iPSCs displayed a rounded shape similar to that of y-iPSCs mitochondria, were more abundant than in y-iPSCs and further increased in number during differentiation (Fig. 1B). On the contrary, a-iPSCs failed to complete the differentiation into motor neurons. In fact, after 15 days (15d) in differentiating condition a-iPSCs did not show neither elongated nor branched neurites, mitochondria formed a convoluted network which occupied most of the cytoplasmic space and cells underwent apoptosis (Fig. 1B, and 2B). These findings suggest that a-iPSCs lost the ability to differentiate properly. One reason that may explain this behavior is that a-iPSCs have already a great number of mitochondria in the proliferation status, which cannot increase further after the trigger to differentiate into neurons. This determines a dysfunctional mitochondrial status that induces iPCSs to initiate apoptosis. It has been reported that mitochondrial dysfunctions lead to cell death [24, 25]. Therefore, by TUNEL assay we measured the percentage of apoptotic cells during differentiation, finding that y-iPSCs had a negligible number of apoptotic cells (<6.5%) throughout the differentiation period, whereas a-iPSCs increase the number of apoptotic cells from 2.6% (at T0) to 34.79% (at 15d) (Fig. 1C and 2). This is in agreement with the arrest of differentiation at 15d in a-iPSCs.

To understand the senescence-related mitochondrial pathway, we investigated the expression of genes regulating mitochondrial biogenesis. In mammals, the main proteins that regulate this process are the mitochondrial transcription factor A (TFAM), the heterotrimeric polymerase gamma which comprises a catalytic subunit (POLG), and two accessory subunits (POLG2), and the mitochondrial RNA polymerase (POLMRT) which provides RNA primers for the replication of the mitochondrial genome [14, 26]. POLMRT forms a complex with two other mitochondrial transcription B cofactors (TFB1M or TFB2M) that possess also a dimethyltransferase activity and are necessary for mitochondrial gene expression [27, 28].

**Figure 4:**
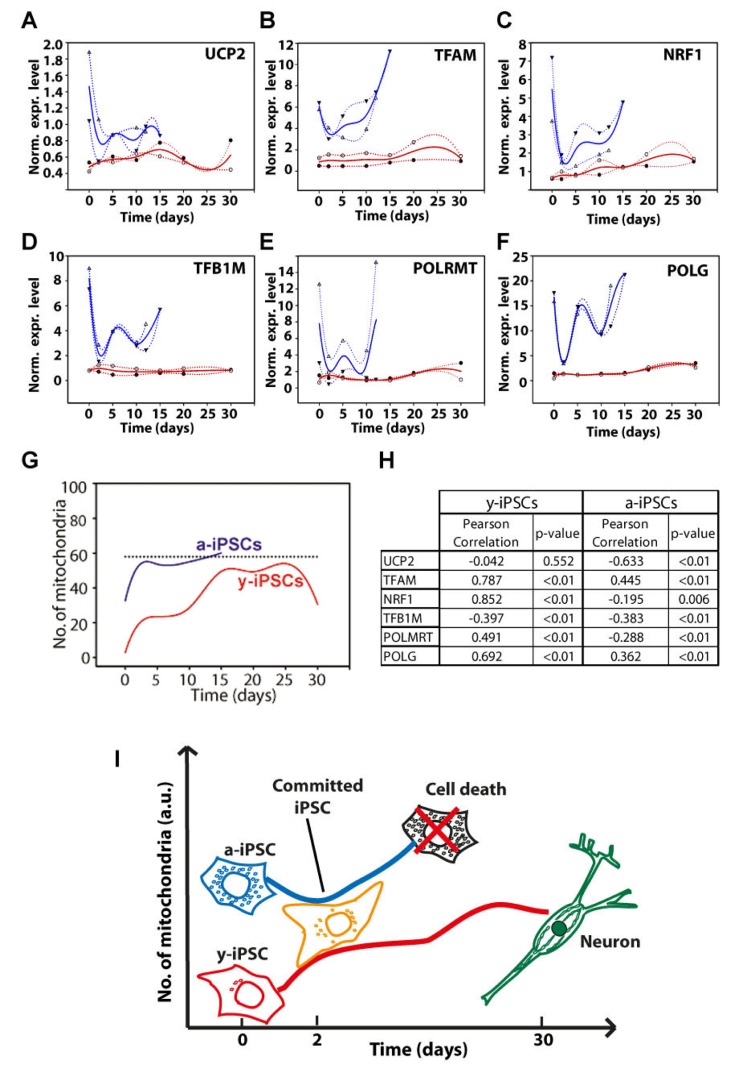
Gene expression analysis of genes relevant to mitochondrial biogenesis and schematic drawing depicting the differences between young- and aged-iPSCs during neuronal differentiation in relation to mitochondrial number and cell death (**A-F**) Normalized expression levels of UCP2 (**A**), TFAM (**B**), NRF1 (**C**), TFB1M (**D**), POLMRT (**E**) and POLG (**F**) genes. Red and blue lines indicate the expression behavior of y- and a-iPSCs, respectively, during differentiation. (**G**) The number of mitochondria, obtained by immunofluorescence, has been displayed for y- and a-iPSCs (red and blue lines, respectively). Dotted line indicates an arbitrary number of mitochondria. (**H**) Correlation between the number of mitochondria and gene expression levels has been calculated and reported in the table together with Pearson's correlation coefficients and statistical significance (p-value). (**I**) Scheme illustrating y- and a-iPSCs in relation to their number of mitochondria (on the y axis) before and during in vitro neurogenesis (with the time reported on the x axis). The black cell depicted, which represents a-iPSCs during neuronal differentiation, is full of mitochondria and encounters cell death.

**Figure 5:**
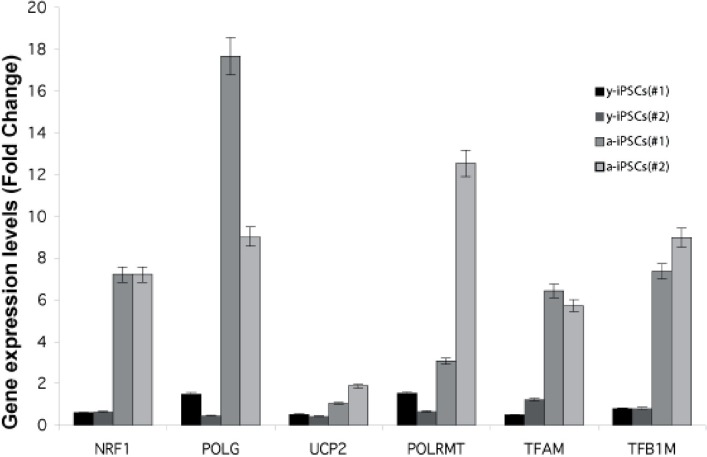
Gene expression analysis of genes relevant to mitochondrial biogenesis in young and aged-iPSCs at day 0 Expression level of NRF1, POLG, UCP2, POLRMT, TFAM, TFB1M genes for two biological replicates (#1, #2) of y- and a-iPSCs. Data are represented as mean ± SD.

**Figure 6:**
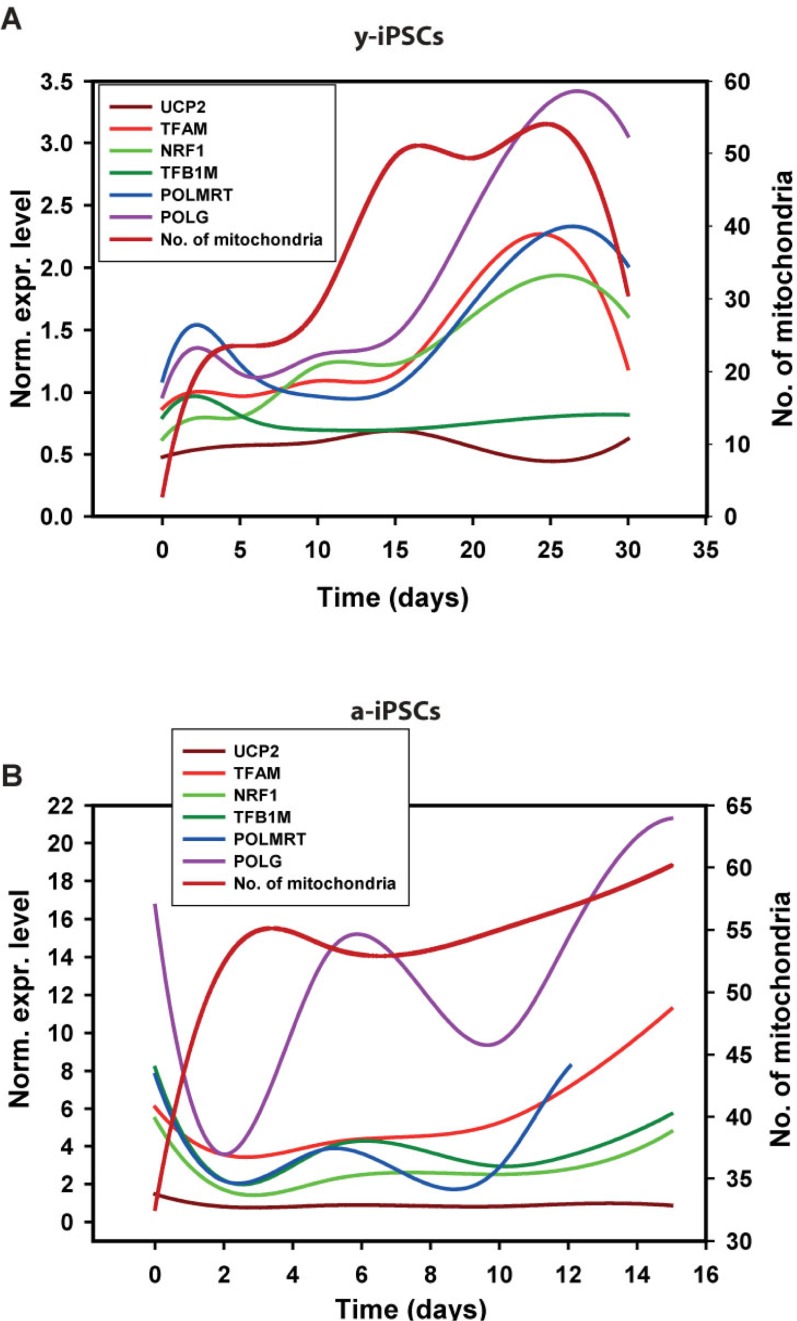
Gene expression analysis of genes relevant to mitochondrial biogenesis in young and aged-iPSCs during neuronal differentiation in relation to the mitochondrial number Normalized expression levels of mitochondrial genes superimposed to the curve obtained after interpolation of mitochondrial number during differentiation of y-iPSCs (**A**) and a-iPSCs (**B**).

We investigated the expression of genes involved in mitochondrial biogenesis (i.e., *UCP2*, *TFAM*, *NRF1*, *TFB1M*, *POLMRT* and *POLG*) during neuronal differentiation of both y- and a-iPSCs to investigate the existence of potential links between the number of mitochondria and the transcriptional control of mitochondria biogenesis. Previous studies demonstrated a high variability amon different murine embryonic stem cell lines [29]. We performed a gene expression study by real-time qPCR in two distinct biological replicates and reported the expression levels of these genes as the trend obtained by spline interpolation of the expression values during differentiation (instead of reporting the mean value as a function of time, Fig. 4). We displayed the normalized expression values, meaning that the absolute amount of starting mRNA for each gene (at T0) in both y- and a-iPSCs has been quantified and taken into account as starting reference point (Fig. 5). Of note, a-iPSCs generally show a higher expression of all of the genes compared to y-iPSCs and an increased expression levels of all these genes at 2d in y-iPSCs is observed, whereas in a-iPSCs we obtained a marked decrease of the same genes (Fig. 4A-F and 6).

The expression of *UCP2* gene in y-IPSCs show a modest increase up to 15d then decreases again to starting values (Fig. 4A). A modest increase is also observed for a-iPSCs up to 15d. In differentiating y-iPSCs, the expression of *TFAM* shows an increasing trend up to a maximum value at 25d, whereas in a-iPSCs the increase is faster and more sustained compared to y-iPSCs (Fig. 4B). Previous studies reported that this increase is correlated to mitochondrial DNA replication [30]. The nuclear transcription factor-1 (NRF1) acts on the majority of nuclear genes encoding subunits of the respiratory complexes, and it is also involved in the regulation of mitochondrial transcription factors, heme biosynthesis and other proteins required for respiratory function. Moreover, recent studies demonstrated that NRF-1 regulates the expression of TFB1M [31]. In y-iPSCs there is a continuous but periodical increase in the expression of these genes during differentiation (Fig. 4C, D). Similarly, after the initial decrease at 2d, in a-iPSCs the expression seems to oscillate although the increase is again more pronounced up to 15d where the expression level returns to the starting values. The expression of *TFB1M* in y-iPSCs did not display significant variations during differentiation, whereas in a-iPSCs the level of expression displayed significant variations in a very limited time period (Fig. 4D). Similarly to *TFB1M*, also the expression of *POLRMT* in y- and a-iPSCs followed a similar behavior (i.e., high differential expression in a limited period of time) (Fig. 4E). Their expression is in agreement with their role in mtDNA transcription that requires the simultaneous presence of POLMRT and the auxiliary factors TFB1M for promoter recognition [32]. In y-iPSCs, *POLG* expression was relatively stable until the terminal phases of neuronal differentiation, where a modest increase was observed (Fig. 4F). Interestingly, this is in line with the reported increased expression of POLG during mtDNA depletion [33]. In a-iPSCs *POLG* expression is again periodic but tend to increase at 15d (Fig. 4F).

Next, we investigated if these expression data could correlate with the increased number of mitochondria. Therefore, we quantified the number of mitochondria in y- and a-iPSCs and performed a correlation (Pearson's) analysis (Fig. 4G, H). Interestingly, in y-iPSCs the expression values for all genes, but *UCP2*, significantly (p<0.01) and directly correlate with the increasing number of mitochondria during differentiation (Fig. 4H and 6). Only *TFB1M* inversely correlate to it. In a-iPSCs, all of the genes are significantly (p<0.01) inversely correlated with the number of mitochondria except *TFAM* and *POLG* that are directly correlated. Noteworthy, the only two genes that are positively correlated with the number of mitochondria in both y- and a-iPSCs are *TFAM* and *POLG* that displayed also the greatest gene expression level at d25-30 for y-iPSCs (Fig. 4B) and d15 for a-iPSCs (Fig. 4F and 6).

Overall, our work suggests that the marked oscillating gene expression behavior of a-iPSCs during differentiation and their great number of mitochondria compared to y-iPSCs, reflects their intrinsic instability and failure to differentiate. To this regard, at d2 the expression level of all the genes decreases at levels similar to those of y-iPSCs that, on the contrary increases from T0 to d2. This suggest that a-iPSCs and y-iPSCs are committed to neuronal differentiation in a very similar way but that, owing to the greatly altered mitochondrial endowment of a-iPSCs, the reestablishment of a correct cell differentiation asset fails, thus driving the cells to cell death (i.e., apoptosis) (Fig. 4I). We hypothesize that cell death is linked to the relative number of mitochondria during differentiation and that after a defined threshold level (Fig. 4G), iPSCs fail to differentiate and undergo apoptosis. We found that a-iPSCs are more prone to this phenomenon owing to their high constitutive number of mitochondria compared to y-iPSCs.

**Figure 7:**
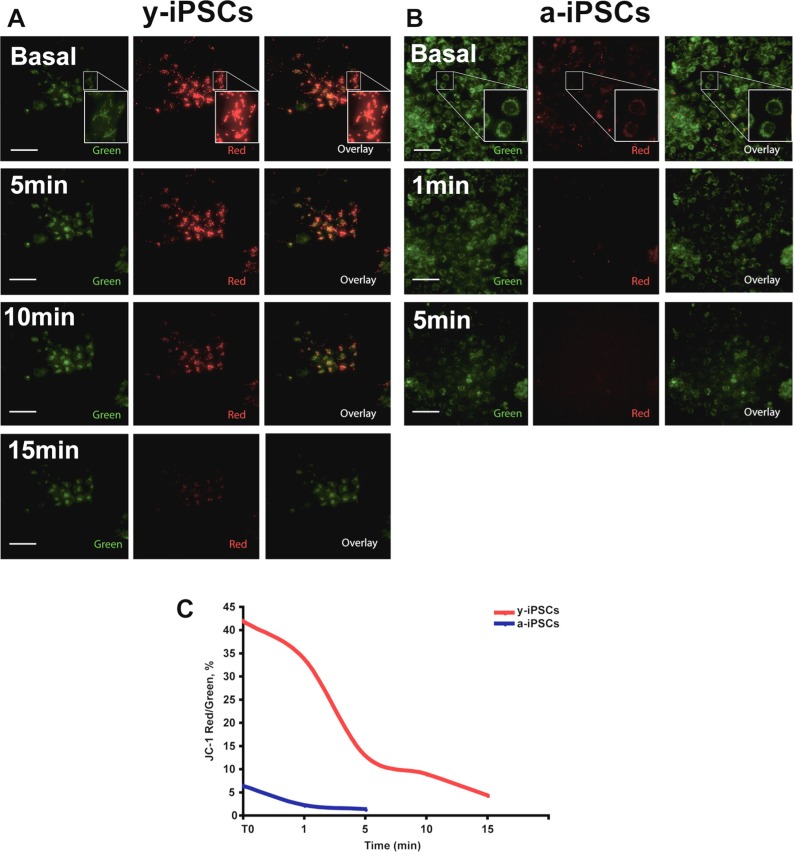
y- and a-iPSCs present differences in basal MMP and following H_2_O_2_ exposure (**A**) JC-1 staining images before and after 5, 10 and 15 minutes after H_2_O_2_ exposure in y-iPSCs. (**B**) JC-1 staining images before and after 1 and 5 minutes after H_2_O_2_ exposure in a-iPSCs. Regions of high mitochondrial polarization are revealed by red fluorescence due to J-aggregate formation of the concentrated dye, whereas depolarized regions are indicated by green fluorescence of JC-1 monomers. Pictures are representative images chosen among 3 independent experiments performed in live imaging condition where CO_2_ (5%) and Temperature (37°C) were controlled using a top stage incubator. Magnified views of the boxed area are displayed as Insets in A and B. Scale bar: 75 μm. (**C**) Time course of y- and a-iPSCs before and during H_2_O_2_ treatment showing the fluorescence intensity ratio (expressed as %) for JC-1 staining (Red/Green fluorescence intensity ratio).

To determine the mitochondrial activity in y- and a-iPSCs, we measured their mitochondrial membrane potential (MMP) in basal condition and after H_2_O_2_ exposure by JC-1 staining (Fig. 7). JC-1 is a cationic dye that presents potential-dependent accumulation in mitochondria and indicates their polarization by a fluorescence emission shift from green (525 nm) to red (590 nm). In particular, regions of high mitochondrial polarization are revealed by red fluorescence due to the J-aggregate formation of the concentrated dye, whereas depolarized regions are indicated by the green fluorescence of JC-1 monomers. Therefore, JC-1 staining allows to monitor mitochondrial depolarization, which is indicated by a decrease in red/green fluorescence intensity ratio. We found that MMP in basal condition was decreased in a-iPSCs when compared to that of y-iPSCs (Fig. 7). Moreover, we monitored JC-1 staining following exposure to H_2_O_2_ and observed a progressive loss of red J-aggregate fluorescence in y-iPSCs from 5 to 15 minutes (Fig. 7A). On the contrary, under basal condition the red J-aggregate fluorescence of a-iPSCs was less intense than that of y-iPSCs and the observed fluorescence decrease was faster (~1 minute after H_2_O_2_ treatment), and disappeared after 5 minutes (Fig. 7B). In conclusion, these results emphasized that H_2_O_2_-dependent MMP depolarization in a-iPSCs is faster than that observed in y-iPSCs and, that the different ability of y- and a-iPSCs to counteract oxidant exposure is linked to their different MMP under basal condition.

## DISCUSSION

It is generally acknowledged that iPSCs can be maintained and propagated indefinitely in culture. However, knowledge on iPSC aging is still insufficient to accept unconditionally this concept. Therefore, we aimed at furthering the knowledge of iPSC biology in relation to aging. Among the hallmarks of aging, mitochondria have a fundamental role. In fact, mitochondria participate in energy production, calcium homeostasis and maintenance of membrane potential. A fine regulation of mitochondrial number and maturity level is required for the proper cellular differentiation, and in particular for neuronal differentiation where mitochondria are involved in the regulation of axonal/dendritic transport and release/reuse of neurotransmitters. Indeed, given the mitochondrial involvement in stemness and differentiation, one can ask whether manipulating mitochondrial content and/or their function, or modulating specific mitochondrial-related signaling pathways, can be used for a more efficient generation or differentiation of iPSCs, that are two crucial aspects in regenerative medicine [34].

Our data demonstrate that maintaining iPSCs indefinitely in culture has strong consequences for their mitochondrial endowment. The higher is the number of culturing passages *in vitro* the higher is also the number of mitochondria, which affects their differentiation potential into neurons afterwards. Noteworthy, prolonged culturing can lead to accumulation of genetic mutations, and we do not exclude that this has occurred in our culturing conditions. The cells with increased number of mitochondria, potentially driven by an altered genetic status were, therefore, positively selected with prolonged culturing, probably because they were advantageous to iPSCs. This possibility does not exclude, and instead confirms that iPSCs maintained in culture for long periods change their biological features and that in the commonly used culturing conditions, the cells with increased mitochondrial number are enriched. It is well acknowledged that prolonged culturing can lead to genetic instability and that, despite the generation of iPSCs is supposed to reset their epigenetic status, some epigenetic modifications were observed in prolonged culturing conditions [5]. Noteworthy, different research groups have reported cases of variable X chromosome inactivation in iPSCs obtained from female subjects [35, 36, 37]. Therefore, to avoid variations due to different status of X inactivation, we used male-derived iPSCs for this study. iPSCs present epigenetic changes in different regions of the genome and, in particular, *in vitro* culturing may lead to DNA methylation alterations [38, 39]. Thus, all these considerations suggest the necessity to carefully consider and avoid genetic instabilities when considering iPSCs for therapeutic applications. The number of mitochondria is directly dependent on the biogenesis of these organelles, on their degradation and on the autophagic digestion (i.e., mitophagy) of the organelles themselves [40]. Moreover, a defective oxidative phosphorylation leads to mitochondrial degradation, whereas an increased mitochondrial stress leads to cell death by apoptosis [24, 25]. In fact, the cellular accumulation of old and damaged mitochondria has been reported to determine age-related dysfunctional mitochondrial turnover [41]. Moreover, the mitochondrial mass is increased in aging cells [42]. Therefore, a-iPSCs represent the condition of aged stem cells that have lost their pluripotency potential, despite maintaining their self-renewal properties. In this work, we demonstrated that iPSCs age in culture, that mitochondrial number and morphology are indicators of the iPSC age, and that the amount and status of mitochondria affect the differentiation potential of iPSCs. In addition to this, the measurement of the MMP indicated that the mitochondria of a-iPSCs are i) less polarized when compared to that of y-iPSCs and ii) they respond to H_2_O_2_ exposure following fast depolarization kinetic. These data demonstrate that the physiology of mitochondria in a-iPSCs is jeopardized, together with altered mitochondrial number and biogenesis.

Undoubtedly, the advancing technology of iPSCs offers a novel and appealing approach to investigate the mitochondrial function as well as mitochondria-related diseases, in particular for the developmentally regulated neurologic diseases. However, the mitochondrial biology of iPSCs needs to be more carefully understood prior to employing iPSCs as innovative and effective therapeutic tools in many human diseases. For these reasons, we deem essential a throughout understanding of the crosstalk between mitochondrial dynamics, biogenesis, mitophagy and stem cell self-renewal and differentiation to fully unravel the etiology and the wide heterogeneity of pathologies associated with mitochondrial dysfunctions.

It is well known that stem cells contain few mitochondria and rely mainly on glycolysis for their energy demand, while differentiated cells present a well-developed mitochondrial network and rely on oxidative phosphorylation for their energy production [43]. Interestingly, Wanet et al. [34] suggested that mitochondrial changes during differentiation would also contribute to differentiation itself, thus supporting the existence of an interplay between mitochondrial biogenesis and stem cell differentiation.

This data on y- and a-iPSCs, together with the background literature mentioned in the introduction, suggest that prolonged time in culture leads to adaptation of the iPSCs to the cell culture environment (which does not mimic the physiological and anaerobic stem cell niche), consequently driving to increased number of mitochondria, disrupted differentiation potential and, interestingly, these features resemble characteristics of aged stem cells. Noteworthy, the results obtained in this study advance the current knowledge of iPSC biology in consideration of their potential use in regenerative medicine. The knowledge deriving from this study will be used in the future to design an *in vitro* environment that mostly resemble that of the physiological stem cell niche, able to preserve the integrity and functionality of stem cells *in vivo*. Therefore, we consider necessary to further understand how to properly handle iPSCs and their environment, thus speeding the pace toward the application of iPSCs in the field of therapeutic applications.

Overall, four main consequences arise from our results that merit further investigations. First, a-iPSCs can be considered a model to study cell aging in culture; second, reprogramming cells from patients with mitochondrial disease may affect the reprogramming efficiency; third, iPSCs obtained from patients with mitochondrial disease may have normal phenotype and differentiation potential owing to the choice of differentiated cells with a healthy mitochondria endowment (i.e., mitochondrial heteroplasmy), similarly to what has been observed in muscle satellite cells and myoblasts from patients with progressive myopathies due to mitochondrial DNA mutations [44]; fourth, the defective mitochondrial phenotype of iPSCs may arise only after a definite culturing passage as a result of the increased mitochondrial number. These observations suggest that modeling mitochondrial diseases with iPSCs require a careful monitoring of their mito-chondrial status before and after reprogramming, during maintenance in culture, and a comprehensive analysis of their differentiation potential. Further studies will be necessary to fully understand: 1) if mitochondria have control over reprogramming and differentiation, 2) if their structural changes in the reprogrammed cells and/or during differentiation are involved in these biological processes, and 3) the signaling pathways involved in the mitochondrial biogenesis and mitochondrial functionality of iPSCs. In conclusion, the present work explores issues that have been ignored, related to indefinite culturing of iPSCs and to the iPSC biology of aging. In addition this study poses questions related to the possible future applications of iPSC-technology and related to the currently poor knowledge of stem cell aging, thus addressing both technical and conceptual challenges of modern science.

Understanding the biological processes controlling stem cell proliferation, cell differentiation and age-associated stem cell dysfunctions is a crucial information needed to fully exploit the potential of pharmacologic manipulations with stem cells, that can slow, and hopefully reverse, age-related degenerative alterations. In fact, mitochondrial alterations are characteristic of several neurodegenerative human disorders, such as Parkinson's, Alzhemier's and Huntington's diseases [45, 46]. Moreover, the potential reversibility of the aging process is a feasible therapeutic avenue suitable not only for age-related disorders, but also for premature aging diseases.

The link between aging and mitochondria has been and still is the field for interesting scientific discussion and many theories related to cell aging have arisen [47]. Accumulation of large defective mitochondria has been associated with cell senescence and aging [48]. In this condition, autophagocytosis is poor and defective mitochondria that are not eliminated are free to replicate without control [49]. Noteworthy, mammalian Target of Rapamycin (mTOR) pathway inhibits autophagy, whereas mTOR inhibitors (i.e., rapamycin) promote autophagy [50, 51]. Therefore, mTOR pathway can be considered as a potential target for pharmacological intervention. In fact, it has been observed that inhibition of mTOR (either by using rapamycin or by depletion of mTOR Complex 1), delayed some characteristics of cell senescence in primary human fibroblasts [52]. Moreover, mTOR pathway regulates mitochondrial biogenesis [53]. This further suggests that the potential role of the mTOR pathway needs to be carefully examined in aging. By inhibition of mTOR pathway, the autophagy process can be re-activated and the control over the quantitative and qualitative features of mitochondria can be re-established.

In conclusion, we think that by continuing the study of stem cell's functioning and aging and the pathways that regulate the aging process itself, it will be possible to better understand the biology of stem cells and to develop innovative therapeutic tools able to change the way we age and alleviate individual age-related diseases and finally to leverage benefits for our aging population, and savings for national healthcare systems.

## METHODS

### iPSC Derivation

Human iPSC lines were purchased from System Biosciences (Cod SC102A-1, USA), obtained from skin fibroblasts of healthy individual and reprogrammed using non-integrating episomal technology (Minicircle DNA and mc-iPS Cells, Euroclone). iPSCs were generated using the minicircle DNA technology (Cod SC301A-1, circular non-viral DNA generated by intramolecular recombination from a parental plasmid mediated by ΦC31 integrase), containing cDNAs of human NANOG, SOX2, OCT4, LIN28 genes in vector as described in [54].

### Cell Culture Conditions

Following thawing, iPSCs were grown on MEFs (Life Technologies) for the first 4-5 weeks and then in feeder free condition using Matrigel (BD Biosciences) in mTeSR1 (Stemcell Technologies). When the iPSCs are 70-80% confluent, they were passaged (using EDTA treatment) 1:4 and transferred to new wells in feeder-free condition and incubated at 37°C, 5% CO_2_, 20% O_2_, the medium were changed every day and the cells split every 3 days.

### Immunofluorescence assay

For immunocytochemistry, cells were fixed with 4% paraformaldehyde for 20 minutes at RT, washed PBS, and blocked with 10% bovine serum and 0.1% Triton X-100. Primary antibodies include anti β-III TUBULIN (T2200, Sigma Aldrich), anti Mitochondria (NB600-556, Novus Biologicals). Secondary antibodies used were conjugated with Alexa 488 or Alexa 555 (Invitrogen) and nuclei were counterstained with 1 μg/ml Hoechst 33342 (Invitrogen). Coverslips were mounted using PBS/Glycerol (1:1), visualized using a confocal microscope Fluoview FV1000 (Olympus) and acquired with the software FV10-ASW Version 2.0.

### *In Vitro* Neuronal Differentiation

iPSC colonies were induced to differentiate in motoneurons according to Compagnucci et al. [55].

### Confocal imaging and image processing

Images were obtained with an Olympus FV1000 confocal laser scanning microscope (FV10-ASW software, version 2.0) equipped with /Ne (543 and 633 nm) lasers, using the 63X oil immersion objective (numerical aperture 1.4). Optical single sections were acquired with a scanning mode format of 1024×1024 pixels, with a pixel size of 0,21 μm. Acquisition of automated-sequential collection of multi-channel images was performed in order to reduce spectral crosstalk between channels. Quantitative analysis of mitochondria were done on cells stained with anti-Mitochondria antibody using Metamorph software (Molecular Dynamics) on single confocal cross sections taken at the nuclear midplane. The boundaries of the mitochondria were manually delineated with the Metamorph drawing tool from Mitochondria-labeled cells and the number of mitochondria was counted from each cell. On average the number of mitochondria was counted from 50 y-iPSCs and from 50 a-iPSCs. The results obtained derive from three independent experiments.

### TUNEL assay

To detect cells committed to death, we used the ‘In situ Cell Death Detection Kit, Fluorescein’ (Cod 11684795910, Roche) following manufacturer's recommendations. The number of cells positive to the TUNEL assay were counted from three independent experiments and represented as a percentage over the total number of cells (counted using the Hoechst-stained nuclei).

### JC-1 staining

To monitor mitochondrial membrane potential (MMP), 5,5,6,6′-tetrachloro-1,1′,3,3′-tetraethylbenzimidazolylcarbocyanine chloride (JC-1) (Cod T3168, Life Technologies) was used (according to manufacturer's instructions). y- and a-iPSCs were grown in proliferating condition and treated with JC-1 at 5 μM for 30 minutes at 37°C. Following the treatment, cells were washed twice with mTeSR1 and photographed before H_2_O_2_ treatment (10 μM) and 1, 5, 10 and 15 minutes after using an inverted Leica DMi8 microscope (Leica, Germany) equipped with the Top Stage Incubator Okolab (Okolab Srl, Italy). The images were acquired using the software Leica LAS-AF 4.5.0. The quantitative analysis was performed using the fluorescence intensity of the red and green signals (following background intensity subtraction) measured by LAS-AF 4.5.0 Leica software.

### Total RNA Extraction

Total RNA has been extracted from two different IPS cell lines (y- and a-iPSCs) with the Total RNA Purification Plus Kit (Norgen Biotech Corp), according to manufactuter's instructions and quantified by NanoDrop 2000 (Thermo Scientific). The integrity of RNA samples has been analyzed by Agilent 2100 Bioanalyzer and the Total RNA 6000 Nano Kit (Vers. II) (Agilent technologies). The run was performed according to manufacturer's instructions. Electropherograms were analyzed using the Agilent 2100 Expert B.02.06 software that includes data collection, presentation, and interpretation functions. A RNA integrity number (RIN) greater than 8.5 has been taken as the value of a good quality RNA.

### Quantitative PCR assays

For mitochondrial gene expression analysis during iPSCs differentiation, six mitochondrial genes (i.e., *UCP2, TFAM, NRF1, POLG, TFB1, POLRMT*) and one control (i.e., *β-ACTIN*) have been chosen and assayed by quantitative real time PCR (qPCR), according to manufacturer's instructions. Total RNA has been reverse transcribed to cDNA using the High-Capacity cDNA Archive Kit (Applied Biosystems) employing random primers. Reverse transcriptase reaction has been performed using 20 μl of total RNA and 4 μl of specific RT primers in a final volume of 40 μl. Reactions were performed incubating samples for 10 min at 25°C, 60 min at 37°C, 5 min at 85°C, and finally cooled on ice. qPCR assays of selected mitochondrial genes have been performed using the SensiMix TM Probe Kit (Bioline) and normalized using *β*-ACTIN as endogenous gene. The following primers (IDT) have been used: TFAM (Hs.PT.56a.4181476.g), NRF1 (Hs.PT.56a.19519028) UCP2 (Hs.PT.56a.15294923), TFB1M (Hs.PT.56a.5451469), POLG (Hs.PT.56a.25946520), POLRMT (Hs.PT.56a.19950680), *β-*ACTIN (Hs.PT.39a.22214847) (Table 1). Relative quantity of mitochondrial genes has been calculated by means of the 2^−ΔΔCt^ method using the SDS software (Ver. 2.1).

**Table 1:** Sequence of primers used for the qPCR detection of mitochondrial genes

Gene Name	Sequence	
**TFAM**	Probe	5′-/56-FAM/CGCTCCCCC/ZEN/TTCAGTTTTGTGTATTT/3IABkFQ/-3′
	Primer 1	5′-GCCAAGACAGATGAAAACCAC-3′
	Primer 2	5′-CGTTTCTCCGAAGCATGTG-3′
**NRF1**	Probe	5′-/56-FAM/ATGGAGAGG/ZEN/TGGAACAAAATTGGGC/3IABkFQ/-3′
	Primer 1	5′-GTCATCTCACCTCCCTGTAAC-3′
	Primer 2	5′-GATGCTTCAGAATTGCCAACC-3′
**UCP2**	Probe	5′-/56-FAM/TGCCCCTGT/ZEN/CTCCAGTTTTTCTCC/3IABkFQ/-3′
	Primer 1	5′-TGCTGATTTCCTGCTACGTC-3′
	Primer 2	5′-ACCGTGAGACCTTACAAAGC-3′
**TFB1**	Probe	5′-/56-FAM/CAGAGAGAC/ZEN/TTGCAGCCAATACAGGA/3IABkFQ/-3′
	Primer 1	5′-CAGAGGTACTGAGCCATAACAG-3′
	Primer 2	5′-GCAGAACTCAGATGACTTTGACT-3′
**POLG**	Probe	5′-/56-FAM/TGCAGATCA/ZEN/CCAACCTCTTGACCAG/3IABkFQ/-3′
	Primer 1	5′-TCATTCAGACCCAGCTTGTAG-3′
	Primer 2	5′-CTTCTGCATCAGCATCCATGA-3′
**POLRMT**	Probe	5′-/56-FAM/AGCTCCTTG/ZEN/AAGGCACCCTGC/3IABkFQ/-3′
	Primer 1	5′-CGCATAGGACAGCAGGTC-3′
	Primer 2	5′-GACATGTACAACGCCGTGAT-3′
**β-ACTIN**	Probe	5′-/56-FAM/TCATCCATG/ZEN/GTGAGCTGGCGG/3IABkFQ/-3′
	Primer 1	5′-ACAGAGCCTCGCCTTTG-3′
	Primer 2	5′-CCTTGCACATGCCGGAG-3′

### Statistical analysis

Statistical comparison between various groups was performed by the one-way analysis of variance (ANOVA) with either least significant difference or Bonferroni post hoc tests, and by the paired Student t test for independent samples, as appropriate, using the SPSS software (version 12.0.2). Comparisons were made between means from several experiments. Differences were considered significant when P values were less than 0.05 (indicated with *). Spline interpolation has been performed using the packages *stats* and *splines* with the open-source software R (Bioconductor [http://www.bioconductor.org/]).

## Abbreviations

iPSCs: induced pluripotent stem cells
a-iPSCs: aged-iPSCs
y-iPSCs: young-iPSCs
